# From a Mott–Anderson Insulator to an Itinerant Metal in LaCo_1–*x*_Ni_*x*_O_3_: Charge Transfer, Spin-State Percolation, and Lattice Control

**DOI:** 10.1021/acs.jpcc.6c01455

**Published:** 2026-05-06

**Authors:** Meng-Jie Huang, Jens Buck, Jagadesh Kopula Kesavan, Kai Rossnagel

**Affiliations:** † Ruprecht Haensel Laboratory, 28332 Deutsches Elektronen-Synchrotron DESY, 22603 Hamburg, Germany; ‡ Institut für Experimentelle und Angewandte Physik, Christian-Albrechts-Universität zu Kiel, 24098 Kiel, Germany; § Institute for Nanostructure and Solid-State Physics, Center for Hybrid Nanostructures, University of Hamburg, 22761 Hamburg, Germany; ∥ The Hamburg Center for Ultrafast Imaging (CUI), 22761 Hamburg, Germany

## Abstract

The perovskite solid solution LaCo_1–*x*_Ni_*x*_O_3_ is a promising candidate for high-efficiency energy conversion, yet the microscopic evolution of its electronic structure across the Ni concentration-dependent insulator-to-metal transition (IMT) remains a subject of debate. Here, we comprehensively investigate the electronic phase diagram by combining element-specific X-ray spectroscopies (X-ray absorption and photoelectron spectroscopy as well as resonant photoemission spectroscopy) with density-functional theory plus Hubbard *U* (DFT + *U*) calculations. We identify a three-stage mechanism for the IMT: (1) a Mott–Anderson insulating phase (*x* ≤ 0.3) governed by disorder-induced localization within narrow impurity bands; (2) a spin-state-assisted percolation (*x* ≈ 0.5) triggered by the formation of high-spin Co^3+^ bridges that interconnect itinerant Ni^3+^ clusters; and (3) a bandwidth-controlled metallic regime (*x* > 0.5) stabilized by the structural straightening of *B*–O–*B* bond angles. By elucidating how Ni-substitution modulates metal–oxygen covalency and the Co/Ni valence manifolds, this work establishes a comprehensive electronic-structure framework. This framework provides valuable insights for optimizing these materials in future energy applications.

## Graphical abstract

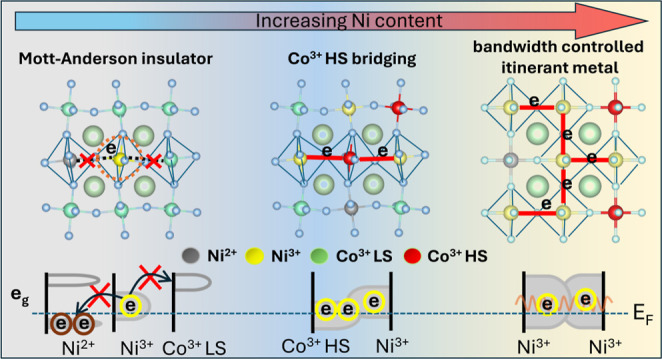

## Introduction

Transition-metal oxides with perovskite structures have attracted considerable attention across condensed matter physics, sustainable energy technologies, and heterogeneous catalysis owing to the intricate coupling among lattice, charge, spin, and orbital degrees of freedom. Within this broad class, the solid solution LaCo_1–*x*_Ni_*x*_O_3_ (LCNO) represents a paradigmatic system for exploring the competition between strong electron correlations and itinerant transport, as it spans the compositional range between two dramatically different end members: insulating LaCoO_3_ (LCO, *x* = 0) and metallic LaNiO_3_ (LNO, *x* = 1). These two end members exhibit fundamentally different electronic ground states. LCO is a strongly correlated (Mott) insulator, in which the ground state arises from a balance between crystal-field splitting (10Dq) and exchange energy (*J*_ex_), giving rise to complex spin-state transitions.^1–9^ In contrast, LNO is an itinerant metal characterized by a wide e_g_ bandwidth and strong p–d hybridization and does not exhibit such spin-state instabilities.^10,11^ The substitution of Co by Ni in LCO provides a continuous tuning parameter for the insulator-to-metal transition (IMT), enabling an evolution from a localized Mott insulator to an itinerant metal.^12–14^

Macroscopic transport measurements have established the presence of an IMT in the LaCo_1–*x*_Ni_*x*_O_3_ solid solution as a function of Ni concentration. At low doping levels (*x* ≤ 0.3), the system largely preserves the insulating behavior of the parent LaCoO_3_, with charge transport commonly described by variable-range hopping (VRH), reflecting strong carrier localization induced by chemical disorder. With increasing Ni content, a crossover toward metallic conductivity emerges within the intermediate composition range (0.3 ≤ *x* ≤ 0.5). The microscopic origin of this transition, however, remains an open question. Although it is often interpreted in terms of ferromagnetic percolation, other detailed investigations have proposed a continuous evolution of the Mott–Anderson insulating state. These observations suggest that a comprehensive understanding of the metallization mechanism has yet to be established.^12,14–18^

Beyond its fundamental interest, the LaCo_1–*x*_Ni_*x*_O_3_ system is technologically relevant for a broad range of electrochemical and catalytic energy applications, including solid oxide fuel cell cathodes and bifunctional oxygen evolution reaction and oxygen reduction reaction electrodes.^19–21^ In this context, the emergence of itinerant charge carriers across the IMT is a prerequisite for sustaining bulk electronic transport during electrochemical operation while simultaneously modulating the density of states near the Fermi level and the e_g_ orbital occupancy—key parameters governing interfacial charge-transfer kinetics.^22–24^ From a fundamental perspective, LaCo_1–*x*_Ni_*x*_O_3_ further provides a paradigmatic platform for investigating the interplay among strong cationic disorder, characteristic of Anderson-type localization, and pronounced electron correlations associated with Mott physics. Unlike *A*-site-doped perovskites, where metallization is often driven predominantly by band filling, *B*-site substitution in this system enables a partial decoupling of disorder effects, correlation strength, and spin-state evolution. Consequently, the absence of a microscopic understanding of how these intertwined electronic parameters evolve across the compositionally driven IMT constitutes a critical bottleneck, limiting the rational design of oxide electrodes whose electron-transfer efficiency can be systematically optimized through band-structure engineering.

In this work, we construct a comprehensive electronic phase diagram of LaCo_1–*x*_Ni_*x*_O_3_. We combine element-specific X-ray photoelectron and absorption spectroscopy (XPS, XAS) and resonant photoemission spectroscopy (RPES) with DFT + *U* calculations to disentangle the cooperative effects of chemical doping, spin-state evolution, and lattice dynamics on the IMT. Our analysis reveals a multistage insulator-to-metal transition, encompassing a disorder-governed Mott–Anderson insulating regime at low Ni concentrations (*x* ≤ 0.3) and, near *x* ≈ 0.5, the formation of percolative conduction pathways mediated by Co^3+^ ions in the high-spin (HS) state that bridge Ni-rich regions, before entering a bandwidth-controlled metallic state (*x* > 0.5) stabilized by the structural straightening of *B*–O–*B* bond angles. These results provide direct spectroscopic evidence for the critical role of spin states in facilitating charge transport in disordered, strongly correlated oxides and establish a microscopic framework for guiding the design of next-generation functional perovskites.

## Methods

Polycrystalline LaCo_1–*x*_Ni_*x*_O_3_ (*x* = 0.0, 0.3, 0.5, 0.7, 1.0), hereafter referred to as LCO, LCN3, LCN5, LCN7, and LNO, respectively, were synthesized by the **Sol–Gel** method.^25–27^ Phase purity and crystallographic structures were confirmed by powder X-ray diffraction (XRD) using Cu Kα radiation (Figure 1a). Representative compositions (LCO, LCN5, and LNO) are presented, with the complete data set provided in the Supporting Information. All samples crystallize in the rhombohedral structure (space group *R*3̅*c*) without detectable impurity phases. The characteristic splitting of the (110)/(104) reflections, highlighted by the shaded region, is clearly resolved for all compositions (see inset), confirming the preservation of the rhombohedral symmetry upon Ni-substitution. In addition, a systematic shift of the diffraction peaks with increasing Ni content is observed, indicating a gradual lattice evolution. The corresponding crystal structure of LaCoO_3_ is illustrated in Figure 1b, where the lattice consists of corner-sharing CoO_6_ octahedra with La ions occupying the *A*-sites. The obtained powder XRD data were analyzed using the **Rietveld** method with the **GSAS-II** software package,^28^ and the detailed results of the XRD refinement analysis, including lattice parameters, bond lengths, and bond angles, are provided in Supporting Information. The observed diffraction peaks are consistent with a refinement in the space group *R*3̅*c* for all substitution levels. To verify the chemical purity of the synthesized samples, wide-range XPS survey scans were acquired using an incident photon energy of 1.5 keV in angle-integrated mode. These spectra confirmed the absence of any detectable extrinsic contaminants within the sensitivity limit of the technique. Further details of sample synthesis, XRD, and XPS survey scans are provided in the Supporting Information.

**Figure 1:**
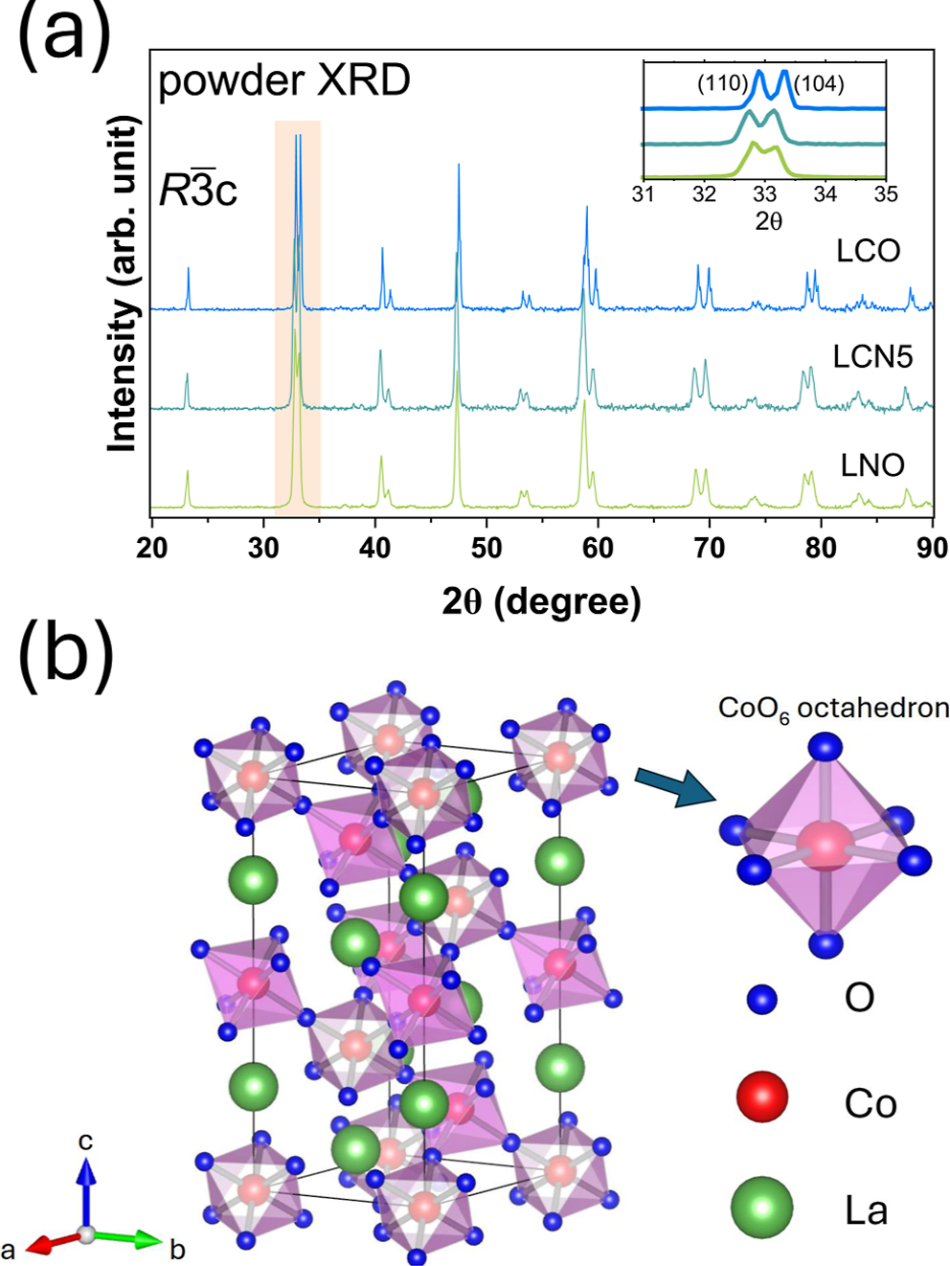
(a) XRD patterns of LaCo_1–*x*_Ni_*x*_O_3_ for selected compositions. All samples can be indexed to the rhombohedral structure (space group *R*3̅*c*). The shaded region highlights the (110)/(104) reflections, whose characteristic splitting is clearly resolved for all compositions, as shown in the inset. (b) Crystal structure of LaCoO_3_ visualized using VESTA software,^29,30^ showing the rhombohedral perovskite lattice composed of corner-sharing CoO_6_ octahedra. La atoms occupy the *A*-sites, while Co atoms are located at the centers of the octahedra. The inset illustrates a single CoO_6_ octahedron.

Core-level XAS and XPS and valence-band (VB) XPS measurements were performed at the ASPHERE III endstation of the P04 beamline at PETRA III (DESY, Hamburg) under ultrahigh vacuum (<1 × 10^–10^ mbar) at a sample temperature of 300 K. Core-level and VB XPS spectra were recorded with an incident photon energy of 1.5 keV and an overall energy resolution of approximately 200 meV. Energy calibration is referenced to the Au Fermi level (*E*_F_) and the 4f_7/2_ core-level peak (at a binding energy of 84.0 eV). RPES was conducted across the Co and Ni *L*_3_ resonances (2p–3d) to individually probe the Co and Ni 3d states.

XAS multiplet calculations were performed within a cluster model incorporating Coulomb interactions (*U*_dd_, *U*_pd_), crystal-field splitting (10Dq), spin–orbit coupling, and ligand-to-metal charge-transfer energy (Δ) between Co/Ni 3d and O 2p states, implemented in the **Quanty** package with a configuration-interaction scheme and managed via the **Crispy** interface.^31,32^

Theoretical partial density-of-states (pDOS) of Co/Ni 3d were calculated using the **WIEN2k** code^33,34^ with the full-potential linearized augmented plane wave plus local orbitals (FP-LAPW + lo) method. To account for the strong correlation among the TM 3d electrons, an effective Hubbard parameter *U*_eff_ = *U* – *J* was employed in the LDA + *U* scheme. In this work, we used *U*_Co_ = 3.0 eV and *U*_*Ni*_ = 4.0 eV for Co and Ni 3d electrons, respectively.^7,35–38^ The 30-atom unit cell (6 La, 6 Co, and 18 O; Figure 1) was adopted as the parent structure, and Ni-substitution was modeled by progressively replacing Co atoms to approximate *x* = 0, 1/3, 1/2, 2/3, and 1. All calculations were performed using lattice parameters refined from the XRD measurements. The calculations were performed using an *R*_MT_ *K*_MAX_ of 7.0 and a 14 × 14 × 5 *k*-mesh. The muffin-tin radii were set to 2.41, 1.91, 1.92, and 1.65 au for La, Co, Ni, and O, respectively.

## Results and Discussion

### Evolution of Valence and Spin States

The evolution of the Co and Ni 2p core-level XPS spectra is presented in Figure 2a,b, respectively. All Co 2p XPS spectra were normalized to the post-edge region at binding energies of 810 eV. On the other hand, owing to the substantial overlap between Ni 2p_3/2_ and La 3d_3/2_ core-level emissions, the Ni 2p XPS spectra were normalized to the intensity of the La 3d_5/2_ peaks, which serves as a stable reference unaffected by Ni-substitution. The results for the parent compounds LCO and LNO are consistent with previous reports, confirming that the valence states of both Co and Ni are +3 and predominantly in the low-spin (LS) configuration.^39–45^ The double-peaked structures located at 836 and 854 eV correspond to the La 3d_5/2_ and 3d_3/2_ core levels, respectively, also indicating the oxidation state of +3 for La in all samples.^46,47^

**Figure 2:**
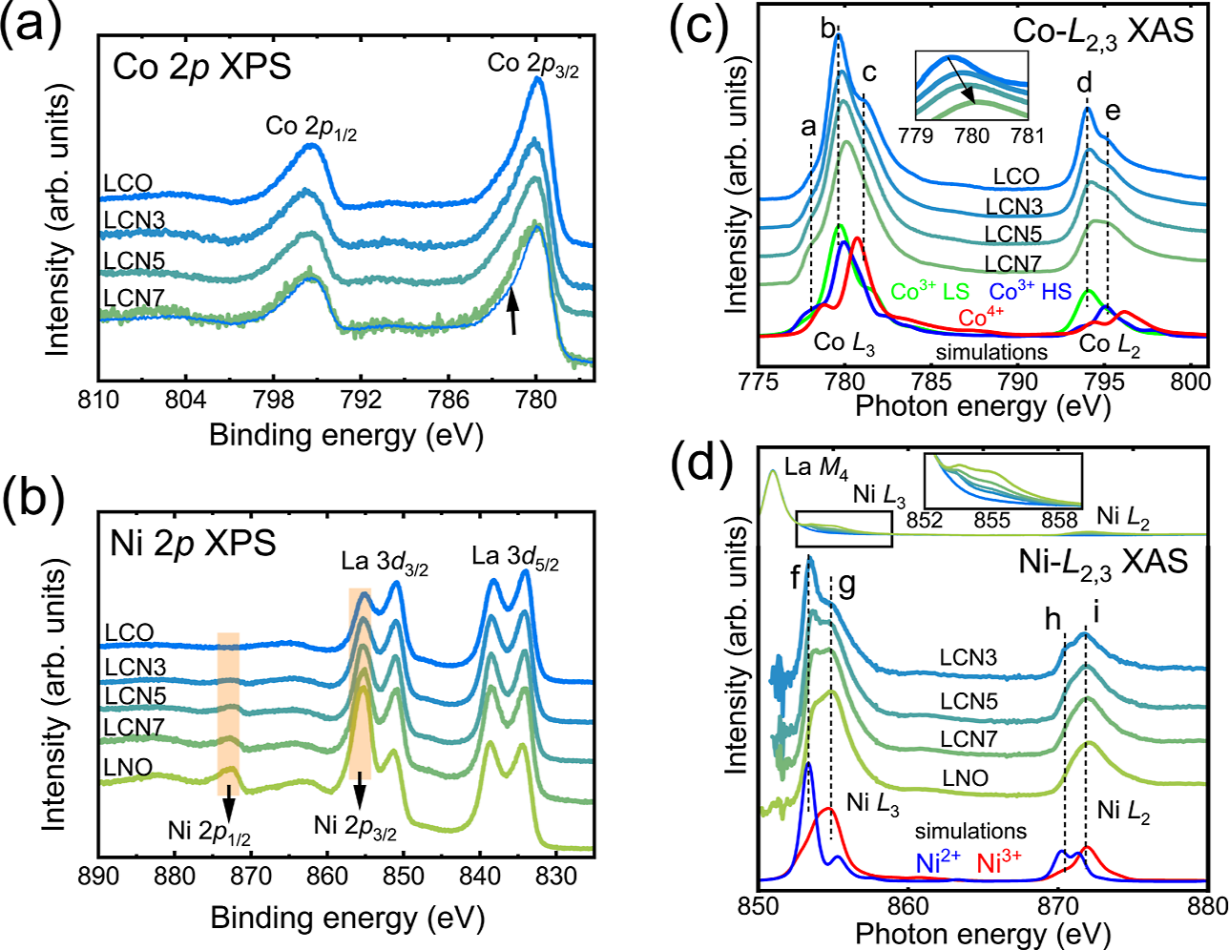
(a) Co 2p and (b) Ni 2p XPS spectra of LaCo_1–*x*_Ni_*x*_O_3_ (LCNO) taken at photon energies of 1.5 keV, respectively. Note that the La 3d signal is also visible in the Ni 2p spectra because the Ni 2p_3/2_ peak overlaps with the La 3d_3/2_ peak due to their close binding energies. (c) Co *L*_2,3_-edge XAS spectra, together with theoretical multiplet simulations for Co^3+^ (LS and HS) and Co^4+^ states. The inset displays the systematic shift of the *L*_3_ peak toward higher energy with increasing Ni content. (d) Ni *L*_2,3_ XAS spectra, together with the theoretical multiplet simulations for Ni^2+^ and Ni^3+^ LS. The top of this figure displays the raw experimental data before the subtraction of the huge La *M*_4_ background. The inset provides a magnified view of the Ni *L*_3_ region, highlighting the evolution of spectral intensity as a function of Ni content *x*. The main lower section presents the net Ni *L*_2,3_ XAS spectra obtained after background subtraction. All 2p XPS and *L*_2,3_ XAS spectra have been vertically offset for clarity.

Overall, the Co and Ni 2p XPS spectra exhibit only minor changes with an increasing Ni content. For Co, a weak shoulder develops on the high-binding-energy side of the 2p_3/2_ peak as the Ni concentration increases (indicated by the black arrow in Figure 2a). This weak shoulder, highlighted by overlaying the spectrum of undoped LCO with that of LCN7, indicates either the emergence of Co^4+^ species or a crossover from Co^3+^ LS to Co^3+^ HS.^42,48,49^ In contrast, the analysis of Ni 2p XPS is complicated by two factors: first, the emission from the Ni 2p_3/2_ core level substantially overlaps with that from the La 3d_3/2_ core level; and second, the intrinsic Ni 2p core-level spectra for Ni^2+^ and Ni^3+^ under *O*_*h*_ symmetry are highly similar.^50^ This combination of spectral overlap and intrinsic line shape similarity makes it difficult to resolve subtle changes in Ni valence with doping.

Next, we examine Co and Ni *L*_2,3_ XAS. Figure 2c,d displays the Co and Ni *L*_2,3_ XAS spectra, respectively, together with the corresponding simulated spectra.^51–56^ All spectra were normalized at an energy 30 eV above the *L*_2_ edge, where the absorption becomes atomic-like and featureless. Unlike the direct overlap between the Ni 2p_3/2_ and La 3d_3/2_ core levels in the XPS, the Ni *L*_3_ in XAS is separated from the La *M*_4_ by approximately 3.5 eV (see the top and inset of Figure 2d). This separation allows us to extract the “net” Ni *L*_2,3_ spectra from the dominant La *M*_4_ background. To obtain the net Ni *L*_2,3_-edge XAS spectra, the La contribution was subtracted from the raw spectra. The pure La *M*_4_ peak was obtained from XAS measured in the Ni *L*_2,3_ energy range on Ni-free LCO, whose crystal structure is very similar to that of LCNO. The resulting difference spectra are plotted in the main lower section of Figure 2d.

The Co and Ni XAS spectra exhibit a clear evolution of the multiplet structure as a function of Ni content. For Co, the progressive growth of the high-energy shoulders (features c and e), combined with the approximately 0.3 eV shift of the *L*_3_ peak (feature b), as highlighted in the inset of Figure 2c, unambiguously signals a continuous increase in the Co^4+^ population with increasing *x*, consistent with the characteristic spectral shift toward higher energies observed for higher oxidation states.^57–60^ Similarly, we also observe a distinct spectral shift toward higher energies for Ni, manifesting as a progressive spectral-weight transfer from the Ni^2+^ multiplet features (f, h) to the Ni^3+^ features (g, i). This observation firmly establishes that the Ni valence state increases monotonically with increasing *x*.^52^

Accompanying the valence-state evolution, a spin-state transition is also observed for Co. As evidenced by the Co *L*_2,3_ spectra, the intensity of shoulder a, located below the *L*_3_ edge, increases with increasing *x*, whereas the intensity of peak d at the *L*_2_ edge decreases. This spectral evolution serves as a characteristic signature of a Co spin-state transition from LS to HS, as corroborated by the calculated Co^3+^ LS and HS reference spectra.^51,56,61,62^

We now quantify the Co^3+^/Co^4+^ and Ni^2+^/Ni^3+^ valence ratios, as well as the evolution of the Co spin-state populations. As discussed above, quantitative determination of valence and spin states based solely on XPS peak fitting is generally less reliable than that based on XAS analysis. Although energy shifts are observed in XPS, interpreting them is complicated by final-state screening effects and Madelung potential variations. Moreover, the quantitative deconvolution of broad core-level spectra into multiple Gaussian or Voigt components often leads to nonunique fitting solutions.^48,49,59,63–66^ In contrast, *L*_2,3_-edge XAS is dominated by atomic multiplet effects that are strictly governed by the local symmetry and 3d orbital occupancy. Thus, XAS serves as a rigorous, local fingerprint of the ground state, allowing for a reliable quantification of the valence and spin manifolds purely based on spectral shape analysis. Therefore, in this study, the valence and spin states of LCNO are determined by fitting the experimental XAS spectra with weighted combinations of atomic multiplet calculations.^56,67–70^

The parameters for the multiplet calculation and the XAS fitting results are provided in the Supporting Information. Figure 3 summarizes the evolution of the valence and spin states as a function of Ni content *x*. Figure 3a shows that the formal valence states of both Co and Ni increase monotonically. The Co valence increases from +3.0 in LCO to +3.3 in LCN7, and the Ni valence rises from +2.69 in LCN3 to +3.0 in LNO. This trend directly reflects the progressive Co-to-Ni intermetallic charge transfer (IMCT) across the series. The spin-state evolution is presented in Figure 3b,c. A gradual spin-state transition of Co^3+^ is identified. The Co^3+^ LS gradually transfers to Co^3+^ HS when *x* ≤ 0.5, that is, the HS fraction increases from approximately 18% in LCO to about 35% in LCN5. Interestingly, the HS fraction becomes nearly constant once *x* exceeds 0.5. The stabilization of the Co^3+^ HS state is closely associated with the local structural relaxation, characterized by the expansion of the Co–O bond lengths and the straightening of the bond angles.^71,72^ Our XRD analysis (see Supporting Information) reveals that for *x* ≤ 0.5, the bond length increases with *x*. This bond elongation reduces the crystal field splitting, thereby promoting the transition from the LS to the HS state. However, for *x* > 0.5, the bond angle becomes the dominant factor driving the spin-state transition. The XRD results for LCN7 show a significant increase in the bond angle. This straighter bond angle reduces the crystal distortion, which enhances the Co 3d–O 2p σ-bonding interaction and broadens the e_g_* bandwidth, thereby stabilizing the Co^3+^ HS.^71,72^ In contrast to the complex spin transition of Co, Ni exhibits a stable spin state and a linear valence shift throughout the series, avoiding crossover due to the dominance of strong crystal-field stabilization energy over Hund’s coupling.

**Figure 3:**
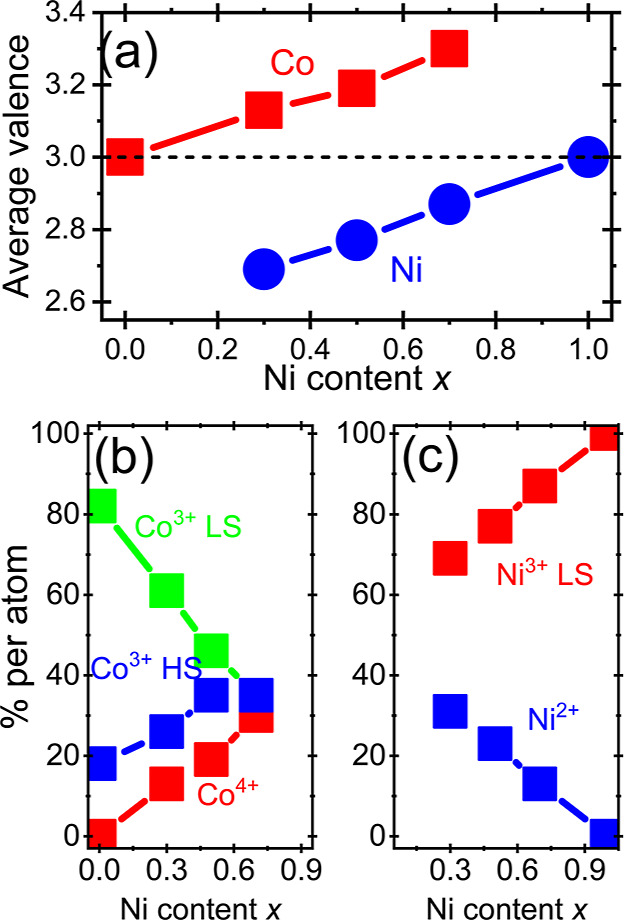
(a) Average valence states of Co and Ni as a function of Ni content (*x*). Panels (b, c) show the evolution of spin state for Co and Ni, respectively.

### Global Electronic Structure Near the Fermi Level

With the valence and spin states of the Co and Ni ions established, we now examine how these local electronic properties collectively shape the density-of-states (DOS) near *E*_F_. Figure 4a shows the VB XPS and oxygen *K*-edge XAS spectra to illustrate the impact of Ni-substitution on the VB and the conduction band (CB), respectively. The Au reference spectrum (black line) is also shown in the XPS data to indicate the position of *E*_F_. For the O *K*-edge XAS, the position of *E*_F_ is determined by comparing the O 1s core-level binding energy of the LCNO sample with the photon energy at the onset of the O *K*-edge absorption.^73–75^

**Figure 4:**
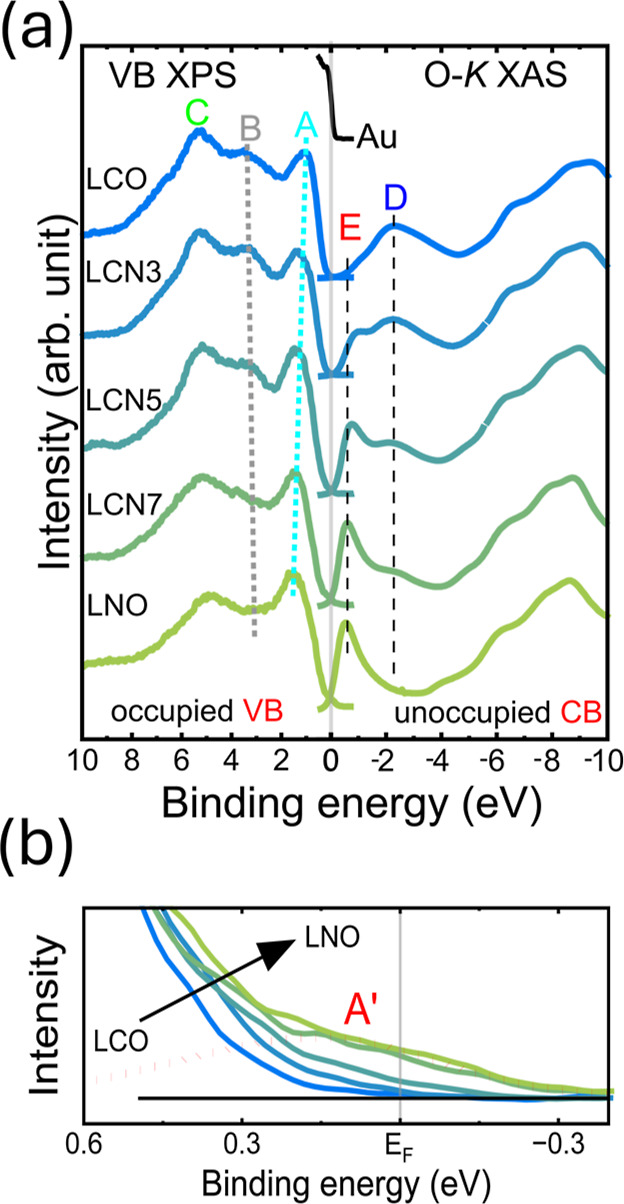
(a) Combined VB XPS and O *K*-edge XAS spectra of LCNO, showing the evolution of occupied (A–C) and unoccupied (D–E) states with Ni-substitution. (b) VB XPS at *E*_F_, highlighting the emergence of feature A′ with increasing Ni content *x*.

The VB consists of three features (A, B, and C), and the CB is composed of two parts (D and E).^3,76–78^ Feature A originates from Co/Ni 3d–O 2p antibonding states; feature B from nonbonding O 2p states; and feature C from Co/Ni 3d–O 2p bonding states. Features D and E, often referred to as the “pre-edge” structure, are attributed to transitions from the O 1s core level to empty O 2p states hybridized with Co/Ni 3d orbitals. This transition is typically described as ${3d}^{n+1}\mathrm{L̲}\to 1s3d^{n+1}$ , where *L* denotes a ligand hole on the oxygen site and the 1s core hole is created by the incident X-ray.

In pure LCO, feature A is primarily attributed to the fully occupied t_2g_* states of Co^3+^ LS (spin configuration: t_2g_^6^e_g_^0^), and it constitutes the highest occupied state in the VB. The bottom of the CB is defined by the empty Co 3d e_g_* states, which are observed as feature D. A finite energy gap of approximately 1 eV is clearly resolved between the t_2g_* (feature A) and e_g_* (feature D) states, consistent with the insulating nature of LCO. With increasing Ni content we observe: (1) Peak A shifts to higher binding energy and feature C (deep bonding) loses intensity; (2) a new feature (A′) appears at *E*_F_ and grows with *x* (indicated in Figure 4b); (3) in the CB, spectral weight transfers from feature D to feature E. The shift of peak A is primarily due to an upward shift in the chemical potential, reflecting a correlated redistribution of d-electrons involving IMCT, mixed-valence states, and enhanced metal–oxygen covalency associated with Ni-substitution. Simultaneously, this shift is accompanied by the significant suppression of feature C, indicating an enhanced TM-O covalency. The loss of feature C’s intensity indicates that electrons are moving from O 2p orbitals to the TM sites, thereby creating ligand holes on the oxygen sites. In the Zaanen–Sawatzky–Allen framework, this implies a small or negative charge-transfer energy (Δ), which stabilizes the high-valence Ni^3+^ and Co^4+^ ions. Consequently, the distinct broadening of feature E near *E*_F_ reflects the increasing bandwidth and itinerant character of these ligand holes, resembling the metallic profile of LaNiO_3_. This is consistent with the formation of ligand holes driven by the small or negative charge-transfer energy (Δ) of high-valence Ni^3+^ and Co^4+^ ions. Moreover, the increasing intensity of feature A′ at *E*_F_, combined with the transfer of spectral weight from feature D to feature E in the CB as *x* increases, provides direct spectroscopic evidence of the IMT.

### Element-Specific Electronic Structure Near the Fermi Level and the Insulator-to-Metal Transition

While standard XPS and XAS capture the overall spectral evolution across the full compositional series, isolating the specific orbital contributions underlying these electronic changes requires an element specificity. To achieve this, we performed RPES measurements on LCNO to separate the Co and Ni 3d partial DOSs. RPES exploits resonance to selectively enhance the 3d-photoemission signal from each element, which is not possible in standard XPS. By tuning the photon energy to the Co or Ni L_3_ absorption edge, one can selectively isolate the Co 3d and Ni 3d contributions to the VB.

To disentangle the overlapping Co and Ni spectral features, we performed RPES measurements across the *L*_3_ absorption edges. Figure 5a,b shows the resonant photoemission intensity maps for LCN5 as a representative example. These maps reveal two distinct spectral behaviors: constant binding-energy stripes corresponding to the Co/Ni 3d valence states and linearly dispersive features arising from the *L*_3_ *M*_4,5_ *M*_4,5_(*LMM*) Auger decay.

**Figure 5:**
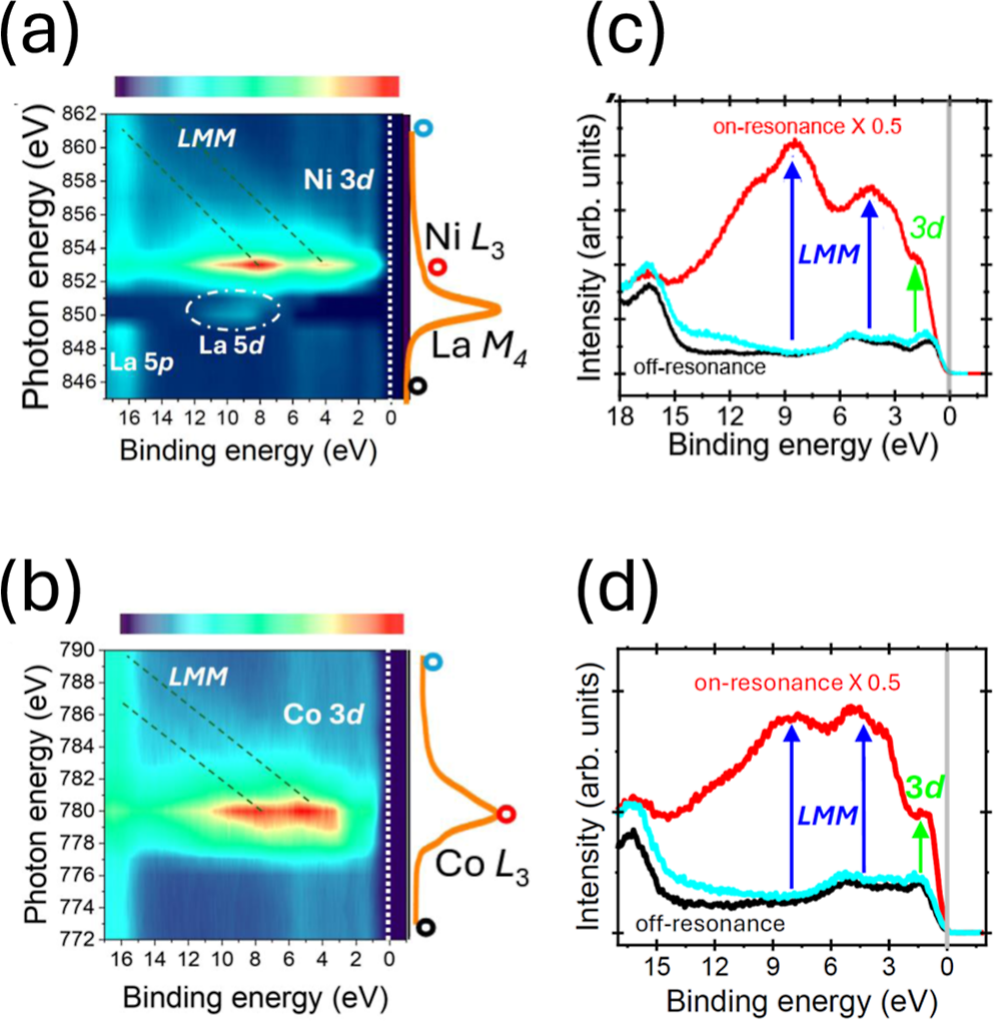
RPES maps of LCN5 measured at the (a) Ni and (b) Co *L*_3_ resonant edge, together with selected VB spectra at representative photon energies (c, d), highlighting the evolution of 3d resonance and *LMM* Auger features. The colors of spectra correspond to the open circles marked on the XAS curves at the respective photon energies.

Notably, in the Ni RPES measurements (Figure 5a), a weak but discernible spectral enhancement appears at the La 3d → 4f resonance (∼850.5 eV). This feature is attributed to La 5d states originating from deep La–O bonding orbitals, indicating that the La cations are partially hybridized with the electronic framework rather than remaining purely ionic.^79^

Guided by the XAS spectra alongside the RPES maps, we selected three representative excitation energies to track the spectral evolution: an “off-resonance” energy in the pre-edge region, an “on-resonance” energy at the *L*_3_ absorption maximum, and a “post-resonance” energy well above the edge. Figure 5c,d shows the VB spectra measured at these photon energies. Although the on-resonance excitation (red curves) yields the strongest signal, it is heavily distorted by overlapping *LMM* Auger emission. Crucially, in the post-resonance regime (cyan curves), the Auger signal shifts to higher binding energy, exposing the intrinsic VB features. This allows us to obtain the pure Co and Ni 3d partial DOS by subtracting the non-resonant background (black curves) from these Auger-free post-resonant spectra (cyan curves).

Figure 6a presents the individual Co and Ni 3d bands extracted from RPES, together with the corresponding partial DOS calculated by DFT + *U* in Figure 6b. We first examine the parent compounds to identify the intrinsic spectral signatures. In pure LCO, the Co 3d bands are characterized by a sharp peak at ∼0.8 eV, corresponding to the fully filled antibonding t_2g_* states. In LNO, the t_2g_* state is located at a higher binding energy (∼1.5 eV), reflecting the higher electronegativity of Ni and the stronger Ni 3d–O 2p covalency. Furthermore, LNO exhibits a distinct spectral weight extending across *E*_F_, identified as the partially filled e_g_* bands which corresponds to feature A′ observed in the VB XPS spectra. Our DFT + *U* results for the parent compounds LCO and LNO reproduce the experimental spectra well, confirming the accuracy and reliability of the DFT + *U* approach.

**Figure 6:**
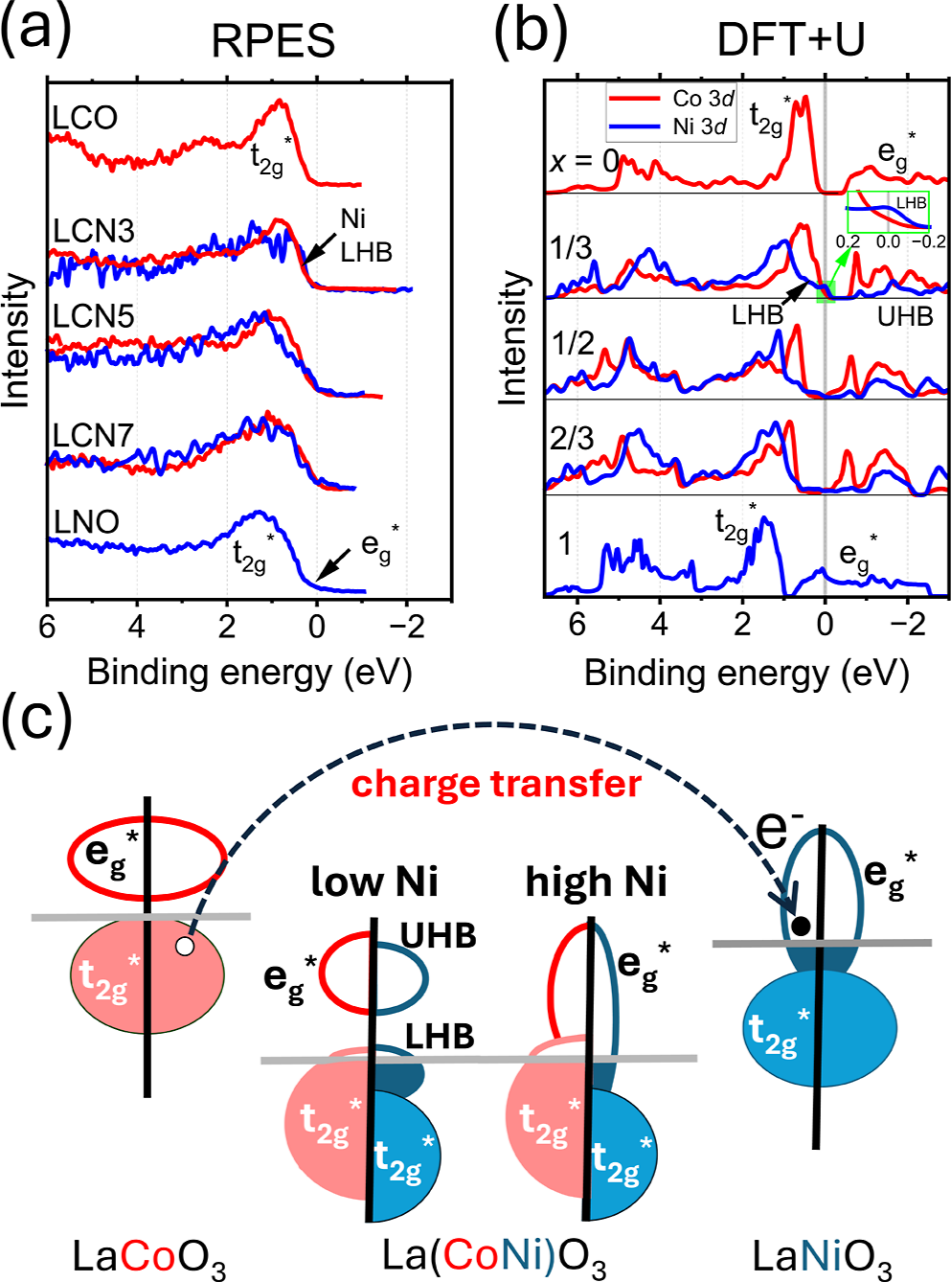
(a) pDOS of Co and Ni 3d states derived from RPES compared with the (b) DFT + *U* calculations for LCNO. (c) Schematic illustration of IMCT and 3d-state evolution in LCNO, showing the transition from localized Hubbard-split Ni e_g_* states to an itinerant CB at high Ni content.

We now turn to the intermediate compositions. At low Ni concentrations (e.g., LCN3), nearly half of the Ni^3+^ (t_2g_^6^e_g_^1^) ions receive an extra electron from Co and are reduced to Ni^2+^ (t_2g_^6^e_g_^2^) due to the Co-to-Ni IMCT. As a result, the originally itinerant Ni e_g_* states become localized. In this regime, the broad e_g_* band is not merely narrowed by disorder but is fundamentally split into lower and upper Hubbard bands (LHB and UHB) by the cooperative effects of on-site Coulomb repulsion (*U*_c_) and *J*_ex_. In particular, while *U*_c_ disfavors double occupancy, *J*_ex_ locks the electrons into a Ni^2+^ HS configuration, thereby suppressing charge fluctuations and reinforcing the Mott–Anderson insulating state.

RPES provides direct evidence for this Hubbard splitting, which is otherwise obscured by the dominant Co signal in standard XPS. By extracting the element-specific Ni 3d states, we unambiguously resolve the LHB feature at ∼0.5 eV. Its precise alignment with the DFT + *U* calculation serves as experimental proof of the correlation-driven band splitting. DFT + *U* calculations clearly identify the LHB and UHB: the LHB lies at the top of the Ni VB with finite spectral weight crossing *E*_F_, whereas the UHB is located about 0.5 eV above *E*_F_. The Co 3d states are also modified by the IMCT and the partial LS-to-HS transition. The combined band tail of the Ni LHB and the induced Co t_2g_* holes is predicted to produce a finite spectral weight slightly crossing *E*_F_ (as shown in the inset of Figure 6b). Experimentally, however, LCN3 remains insulating, with charge transport governed by VRH, in contrast to the metallic behavior predicted by DFT + *U* calculations. This discrepancy likely originates from the intrinsic limitation of periodic supercell models, which tend to underestimate the nonperiodic diagonal disorder (Δ*V*) characteristic of solid solutions. The dominant role of disorder-induced localization is consistent with the transport results reported by Kumar et al.,^12^ who demonstrated the presence of Anderson localization in this regime. While their *K*-edge analysis supported a rigid homovalent substitution model (Co^3+^/Ni^3+^), our element-specific *L*-edge spectroscopy provides an important refinement. It reveals that the disorder potential is not purely chemical but is electronically enhanced through IMCT. The resulting mixed-valence configuration (Ni^2+/3+^ and Co^3+/4+^) substantially increases the random potential, thereby fulfilling the Anderson localization criterion (Δ*V* ≥ *W*_eff_, where *W*_eff_ denotes the effective electronic bandwidth) and stabilizing a localized, proto-percolative electronic state.

As the Ni concentration increases further, the system approaches a critical percolation threshold. Theoretically, the site percolation threshold for a simple cubic lattice is *p*_*c*_ ≈ 0.31.^80,81^ However, experimental perovskite solid solutions with *B*-site substitution, such as SrRu_1–*x*_Ti_*x*_O_3_ and LaFe_1–*x*_Ni_*x*_O_3_, typically undergo the IMT slightly above this geometric limit (*x*_c_ ≈ 0.35–0.5), reflecting that the simple cubic percolation threshold serves only as a qualitative reference. Such deviations arise from structural distortions, orbital hybridization, and disorder and are consistent with the behavior observed in the present LaCo_1–*x*_Ni_*x*_O_3_ system.^82,83^

Our observation in LCNO follows this trend. In LCN3, the itinerant Ni^3+^ concentration is only ∼20% per formula unit (see Figure 3c), well below both the geometric threshold and similar experimental systems. Consequently, any emerging metallic domains remain isolated by the dominant insulating matrix of Co^3+^-LS and localized Ni^2+^.

Between LCN3 and LCN5, the system remains trapped in this disorder-pinned state despite the increasing Ni content. Even at *x* ≈ 0.4, where the nominal density of active sites approaches the geometric threshold, strong diagonal disorder ensures that the metallic clusters remain Anderson-localized. This behavior is consistent with Hammer et al.’s observation that transport up to *x* = 0.4 is governed by VRH, reflecting a continuous Mott–Anderson transition rather than a sharp percolation onset.^14^ Our analysis reconciles this view: although the system behaves as a Mott–Anderson insulator at *x* = 0.4 due to disorder, extending the compositional range to *x* = 0.5 shows that this is actually a suppressed percolation precursor. As the Ni concentration (and Co^3+^-HS bridging) increases further, the system eventually overcomes this disorder limit.

In LCN5, the system transitions to a robust metallic state. Here, the Ni^3+^ concentration rises to ∼38% per formula unit, physically exceeding the geometric percolation limit. Crucially, this connectivity is reinforced by the spin-state transition in the cobalt sublattice. The total population of transport-enabling ions—including itinerant Ni^3+^, bridging Co^3+^-HS, and hole-doped Co^4+^—increases significantly, establishing a long-range conduction network that bypasses the localized barriers.

The nature of these bridges relies critically on orbital symmetry. The Co^3+^-LS state (t_2g_^6^e_g_^0^) is diamagnetic and insulating, since its empty e_g_ shell causes a symmetry mismatch with the active Ni^3+^ (e_g_^1^) conduction electrons. In contrast, the Co^3+^ HS state (t_2g_^4^e_g_^2^) has populated e_g_ orbitals. Together with the intrinsically itinerant holes from the significant Co^4+^ fraction (∼10% per unit cell), these orbitals provide the symmetry-matched bridges that interconnect the itinerant Ni clusters. The recovery of metallicity is not via magnetic double-exchange but is fundamentally driven by enhanced orbital hybridization. The populated Co e_g_ orbitals (and ligand holes) enable strong overlap with the oxygen 2p states, effectively bridging the Ni sites and establishing a continuous Ni–O–Co–O–Ni conduction network across the lattice.

Beyond the percolation threshold (*x* > 0.5), the system undergoes a fundamental crossover to a bandwidth-controlled regime. In the Ni-rich LCN7, the electronic structure becomes fully delocalized and itinerant, similar to LNO. DFT + *U* indicates that the DOSs at *E*_F_ in LCN7 is comparable to that of LCN5, and XAS shows that the fraction of bridging Co^3+^-HS ions saturates beyond LCN5. This suggests that at high doping, the evolution is driven primarily by structural relaxation and carrier injection. The transition to the fully metallic regime is primarily driven by the structural widening of the bandwidth. This arises from a straightening of the *B*–O–*B* bond angles, which increase from 162.91° in LCN5 to 164.24° in LCN7. According to the tight-binding model, the intersite transfer integral *t*_pd_ ∝ cos­[(180° – θ)/2]; so this bond straightening directly enhances orbital overlap. Importantly, this geometric effect is underpinned by strong hybridization between the transition-metal 3d and oxygen 2p orbitals. Both the dominant Ni^3+^ matrix and the minority Co^4+^ ions exhibit significant covalency. The very negative charge-transfer energy of Co^4+^ $\left(d^{6}\mathrm{L̲}\right)$ promotes robust charge redistribution from oxygen to the metal, indirectly reinforcing the metallic state by maintaining a high density of mobile ligand holes. These holes are efficiently delocalized by the broadened bandwidth, preventing the Co impurities from acting as localization centers. Macroscopically, this crossover is evidenced by a drastic reduction in the Seebeck coefficient and the low metallic resistivity observed for high-Ni compositions.^17^

Figure 6c encapsulates the microscopic mechanism governing the IMT and metallization. In the low-Ni regime, the *U*_c_-driven Hubbard splitting isolates the Ni e_g_ states, confining carrier transport. As the system becomes Ni-rich, enhanced orbital overlap from structural straightening significantly broadens the bandwidth. This broadening eventually bridges the Hubbard gap, merging the localized LHB and the UHB into a continuous CB. Thus, the transition from LCN5 to LCN7 represents a fundamental crossover from a percolation-limited regime, constrained by disorder and connectivity, to a bandwidth-controlled regime, where structural straightening stabilizes a fully itinerant metallic state. In contrast to previous transport studies,^16^ which provided only indirect indications of carrier characteristics, our element-specific spectroscopic measurements directly reveal the microscopic mechanism underlying the transition. The results indicate that metallization is primarily governed by cooperative Co spin-state bridging rather than a simple valence change.

## Conclusion

In summary, we have established a unified microscopic model of the LaCo_1–*x*_Ni_*x*_O_3_ solid solution. Core-level spectroscopies (XPS + XAS) reveal that Ni doping induces a continuous IMCT from Co to Ni. As a result, the average cation valence increases across the series: Ni evolves from a partially localized, mixed-valence state (≈Ni^2.69+^) toward an itinerant Ni^3+^ configuration, while Co simultaneously exhibits both an increase in average valence (Co^3+^ → Co^3.3+^) and a pronounced spin-state crossover.

Furthermore, by combining RPES with DFT + *U*, we show that the IMT in LaCo_1–*x*_Ni_*x*_O_3_ is not driven by simple band filling. Instead, it occurs across three cooperative regimes: (1) a disorder-dominated Mott–Anderson insulating regime, (2) a spin-state-mediated percolation regime, and (3) a bandwidth-controlled metallic regime. Our findings highlight that manipulating the specific interplay between metal–oxygen covalency and lattice geometry is crucial for designing next-generation correlated oxide electrodes.

## Supplementary Material



## Notes

Details of sample synthesis and structural characterization; Rietveld refinement results including lattice parameters, bond lengths, and bond angles; XPS survey spectra confirming chemical purity; parameters and results of multiplet calculations; additional XAS fitting analysis; supplementary spectroscopic data; and supporting discussions (PDF)
