# Supplementary material for: From a Mott–Anderson Insulator to an Itinerant Metal in LaCo1–x Ni x O3: Charge Transfer, Spin-State Percolation, and Lattice Control
Source: J Phys Chem C Nanomater Interfaces. 2026 May 6;130(26):9195–204. doi: 10.1021/acs.jpcc.6c01455 (PMC13339768; doi:10.1021/acs.jpcc.6c01455)
Supplement: Supplementary file 1 [file jp6c01455_si_001.pdf]

Supporting Information for From Mott-Anderson Insulator to Itinerant Metal in $LaCo_{1-x}Ni_xO_3$: Charge Transfer, Spin-State Percolation, and Lattice Control

Meng-Jie Huang,[†] Jens Buck,[†,‡] Jagadesh Kopula Kesavan,[¶,§] and Kai Rossnagel[†,‡]

†*Ruprecht Haensel Laboratory, Deutsches Elektronen-Synchrotron DESY, 22603 Hamburg, Germany*

‡*Institut für Experimentelle und Angewandte Physik, Christian-Albrechts-Universität zu Kiel, 24098 Kiel, Germany*

¶*Institute for Nanostructure and Solid-State Physics, Center for Hybrid Nanostructures, University of Hamburg, 22761 Hamburg, Germany*

§*The Hamburg Center for Ultrafast Imaging (CUI), 22761 Hamburg, Germany*

E-mail:

Sample synthesis

Polycrystalline powders of $LaCo_{1-x}Ni_xO_3$ ($0 \leq x \leq 1$) were synthesized using a conventional sol–gel route. High-purity La_2O_3, Co, and Ni powders were employed as precursors. Prior to synthesis, La_2O_3 was preheated at 800 °C for 8 h to remove adsorbed moisture due to its hygroscopic nature. Homogeneous mixing and controlled gelation were facilitated by metal–ligand complexation using nitric acid and diluted citric acid solutions.

Stoichiometric amounts of pre-dried La_2O_3, Co, and Ni were dissolved in 70 mL of 65% HNO_3 under stirring at 110°C for 1 hour to ensure complete dissolution. Subsequently, 5g of citric acid ($C_6H_8O_7$) dissolved in 30mL of deionized water was added to the mixture. The resulting solution was continuously stirred and evaporated at 110 °C until gel formation occurred, followed by heating at 150 °C for 10 min and 200 °C for an additional 10 min. The obtained xerogel was cooled to room temperature under ambient conditions.

The xerogel was then calcined in air through a two-step process at 250°C for 5 hours and subsequently at 500°C for 10 h, with a heating rate of 250°C h^{-1}, to remove carbonaceous residues. The calcined powders were ground thoroughly and sintered at 800°C for 24 hours with a ramp rate of 250°C h^{-1}. After cooling to room temperature, the samples were milled for 30 minutes and reground to improve homogeneity.

To minimize oxygen deficiency and achieve the desired stoichiometry, the powders were annealed in an oxygen-rich atmosphere at 950 °C for 50 h, cooled to room temperature, and milled again for 30 min before pelletization. The pelletized samples were finally annealed in oxygen at 1000 °C for 72 h and subsequently cooled to room temperature prior to characterization.

Powder X-ray diffraction

Powder X-ray diffraction (XRD) patterns for all $LaCo_{1-x}Ni_xO_3$ samples are presented in Fig. S1. The XRD measurements were performed at room temperature using Cu K_α radiation (8.0478 keV, λ = 1.5406 Å) on a Bruker D8 Advance diffractometer over the 2θ

range of 20°–90°. All diffraction peaks can be indexed to the rhombohedral perovskite phase of $LaCoO_3$, and no secondary phases such as La_2O_3, $La(OH)_3$, NiO, CoO, or Co_3O_4 were detected within the instrumental resolution.

The refined lattice parameters (a, c), average bond lengths, bond angles, unit-cell volumes, and goodness of fit (GOF(χ^2)) obtained from Rietveld refinements are summarized in Table S1. The low reliability factors, absence of impurity or amorphous phases, and low background intensity collectively confirm the high phase purity and crystallinity of the synthesized LCNO samples.

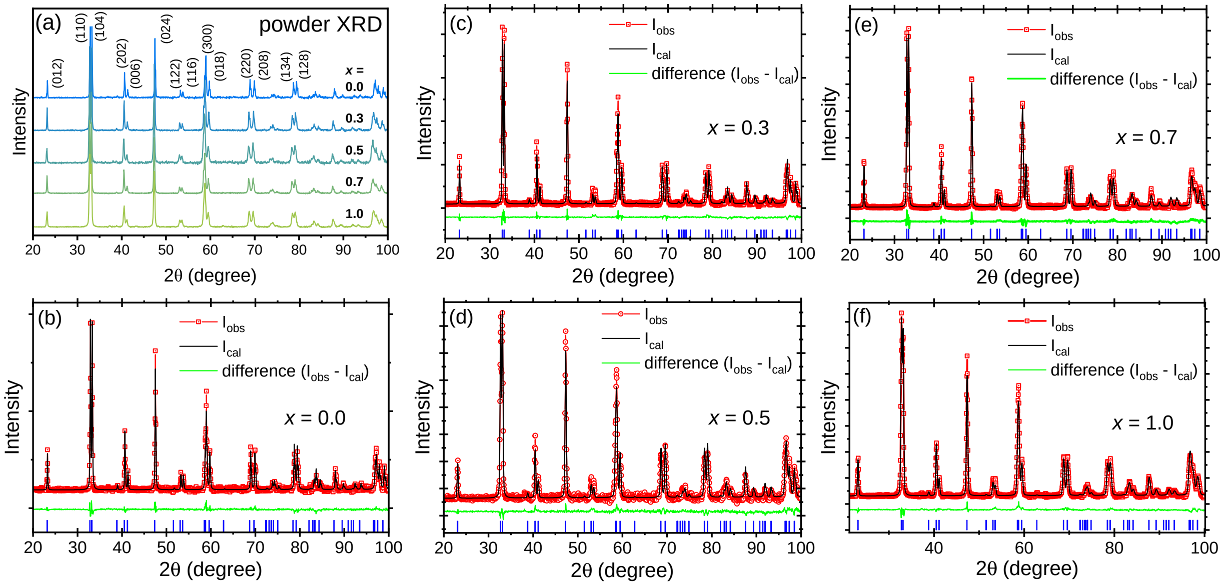

Figure S1: (a) Full powder XRD patterns of $LaCo_{1-x}Ni_xO_3$ for all compositions, including peak indexing and reference reflections used for phase identification. (b) - (f) Rietveld refined XRD pattern for x = 0.0 - 1.0, where the red hollow squares show the experimental data (I_{obs}), black lines represent the calculated spectra (I_{cal}), green lines exhibit the difference (I_{obs} - I_{cal}), and blue bars denote the position of the Bragg peaks.

Table S1: Lattice parameters, volume, bond lengths and angles of $LaCo_{1-x}Ni_xO_3$ derived from the Rietveld refinement of the powder-diffraction data with the space group $R\bar{3}$c.

x	a (Å)	c (Å)	V (Å^3)	d_{B-O} (Å)	θ_{B-O-B} (°)	GOF (χ^2)
0.0	5.4430(5)	13.0961(6)	336.01(4)	1.9243(23)	162.9(3)	1.89
0.3	5.4599(3)	13.1269(5)	338.898(28)	1.9397(9)	163.1(4)	1.98
0.5	5.4630(5)	13.1402(7)	339.62(4)	1.9415(4)	162.91(2)	2.3
0.7	5.4644(5)	13.1498(6)	340.04(4)	1.9385(2)	164.24(8)	2.01
1.0	5.4568(5)	13.1533(6)	339.19(4)	1.9364(10)	164.7(4)	1.94

XPS survey

The X-ray photoelectron spectroscopy (XPS) survey spectra for the $LaCo_{1-x}Ni_xO_3$ samples are shown in Fig. S2. The measurements were performed in an angle-integrated mode with an incident photon energy of 1.5 keV.

The survey spectra reveal all expected constituent elements, including La 3d, Ni/Co 2p, O 1s, La 4p, La 4d, and Ni/Co 3p core levels. Apart from a minor residual carbon contribution arising from the synthesis process, no extraneous peaks associated with impurities or surface contamination were observed. The absence of additional signals confirms the high chemical purity of the $LaCo_{1-x}Ni_xO_3$ samples and the stability of their surface composition.

Analysis of XAS spectra

Table S2 summarizes the parameters employed in the multiplet calculations for the various valence and spin states of Co and Ni. The listed parameters include the crystal-field splitting (10Dq), ligand-to-metal charge-transfer energy (Δ), intra-atomic d–d Coulomb interaction (U_{dd}), core-hole p–d Coulomb interaction (U_{pd}), and the hybridization strengths of the e_g (V_{e_g}) and t_{2g} ($V_{t_{2g}}$) orbitals.

Figures S3(a) and S3(b) show the experimental XAS spectra together with the corresponding multiplet simulation results for Co and Ni, respectively. To illustrate the fitting procedure in detail, we also present the decomposition of the calculated spectrum for the representative composition $LaCo_{0.5}Ni_{0.5}O_3$, in which the total spectrum is reproduced by a

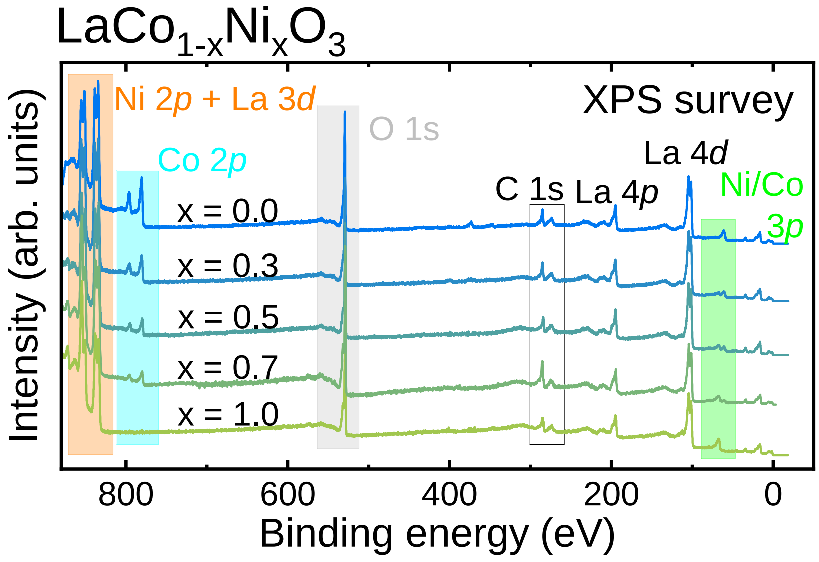

Figure S2: XPS survey scan for $LaCo_{1-x}Ni_xO_3$.

weighted superposition of individual spectral components corresponding to distinct valence and spin states.

Table S2: Parameters for the XAS calculations. The Slater integrals were reduced to 80% of the original Hartree-Fock values for all calculations.

	Δ	10 Dq	U_{dd}	U_{pd}	V_{e_g}	$V_{t_{2g}}$
Co^{4+} IS	-3.0	1.6	5.0	7.0	2.4	1.7
Co^{3+} LS	3.0	1.4	5.0	7.0	2.6	1.1
Co^{3+} HS	3.0	0.8	5.0	7.0	2.5	1.2
Ni^{2+} HS	4.0	1.2	4.0	5.0	2.2	1.1
Ni^{3+} LS	1.0	1.9	4.0	5.0	2.8	1.8

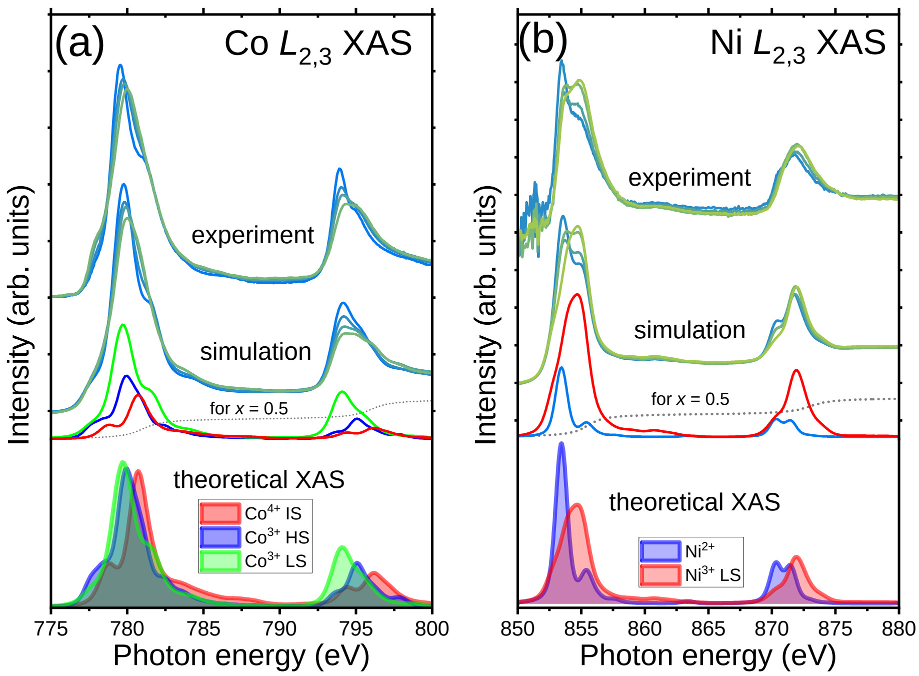

Figure S3: (a) and (b) display, respectively, the experimental XAS for Co and Ni, their corresponding fitting results, a representative fitting for LCN5 as an example (constructed as a weighted sum of different theoretical spectra), and the individual calculated spectra for each specie. The gray dashed lines indicate the edge-jump backgrounds applied during the fitting procedure.
